# Porosity of Solid Electrolyte Interphases on Alkali Metal Electrodes with Liquid Electrolytes

**DOI:** 10.1021/acsami.1c15607

**Published:** 2021-10-20

**Authors:** Kyungmi Lim, Bernhard Fenk, Jelena Popovic, Joachim Maier

**Affiliations:** Max Planck Institute for Solid State Research, Stuttgart 70569, Germany

**Keywords:** solid electrolyte interphase, alkali metal electrodes, liquid electrolytes, battery interfaces

## Abstract

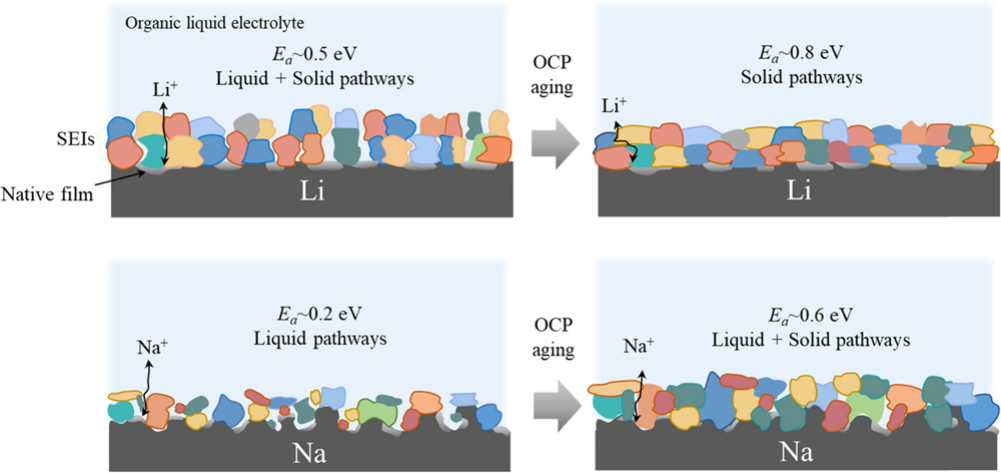

Despite the fact that solid electrolyte interphases (SEIs) on alkali metals (Li and Na) are of great importance in the utilization of batteries with high energy density, growth mechanism of SEIs under an open-circuit potential important for the shelf life and the nature of ionic transport through SEIs are yet poorly understood. In this work, SEIs on Li/Na formed by bringing the electrodes in contact with ether- and carbonate-based electrolyte in symmetric cells were systematically investigated using diverse electrochemical/chemical characterization techniques. Electrochemical impedance spectroscopy (EIS) measurements linked with activation energy determination and cross-section images of Li/Na electrodes measured by ex situ FIB-SEM revealed the liquid/solid composite nature of SEIs, indicating their porosity. SEIs on Na electrodes are shown to be more porous compared to the ones on Li in both carbonate and glyme-based electrolytes. Nonpassivating nature of such SEIs is detrimental for the performance of alkali metal batteries. We laid special emphasis on evaluating time-dependent activation energy using EIS.

## 1 Introduction

Alkali metals find renewed intention as most promising anode materials in battery technology. Among them, Li and Na have exceptionally high theoretical gravimetric capacities (3860 and 1166 mAh g^–1^, respectively) and low redox potentials (−3.04 and – 2.71 V vs the standard hydrogen electrode, respectively), which makes them attractive material options for achieving high-energy-density batteries.^1,2^ However, their electron configuration leads to chemical reactions with the atmosphere in which they are stored and with most electrolytes, resulting in the formation of passivating films on their surface called solid electrolyte interphases (SEIs) in the battery material research community. The physicochemical properties of the SEI play a crucial role in the performance of metal battery cells. Since SEI formation is responsible for the initial capacity loss and poor ionic transport through the SEI causes substantial additional resistance, the general aim is to form a highly ionically conductive as well as electrochemically and morphologically stable SEI.^3^ Therefore, fundamental insights into the mechanism of formation and ion transport in the SEI are crucial for potential utilization of Li and Na metal batteries.^4^

Ever since Peled had first proposed a multicomponent SEI model on anodes upon contact with nonaqueous electrolytes in 1979,^5^ a number of attempts have been made to understand the structure and properties of SEIs using various tools including electrochemical methods,^6−10^ surface-sensitive methods such as X-ray photoelectron spectroscopy (XPS)^11,12^ and infrared spectroscopy,^13^ and various types of electron microscopies.^14^ Based on these experimental investigations, multilayer^7^ and mosaic models^15^ were suggested already in the early stages of SEI discovery. The multilayer model assumes that the SEI consists of several compact/porous layers, allowing for electrochemical impedance spectroscopy (EIS) measurements to be fitted into a series of parallel RC circuits, while the mosaic model suggests a more complex distribution of the organic/inorganic SEI components. Diverse and more advanced experimental/theoretical (e.g., in situ/in operando X-ray characterization techniques, cryo-electron microscopy, atomic force microscopy, liquid secondary ion mass spectroscopy, and ab initio molecular dynamics simulations) tools have recently been introduced, allowing researchers to take a step closer in understanding the nature of SEIs on Li and Na.^16−22^ However, there is still a lack of fundamental understanding of the growth mechanism of SEIs on Li/Na metals, especially under open-circuit potential.^23,24^ The ability to maintain the capacity of batteries under this condition, the so-called shelf life, is a crucial property needed for the utilization of Li/Na metal batteries but is often overlooked. Furthermore, systematic study of the growth/transport mechanism of SEIs on Li vs Na has yet to be done. Recently, the SEI on Na formed by liquid electrolyte has been shown to be less chemically/mechanically stable compared to the SEI on Li^25,26^ and has even been reported to dissolve into specific liquid electrolytes.^25,27^ However, most of the abovementioned claims are based on indirect evidence, without thorough analysis involving combination of electrochemical/chemical/morphological characterization.

In this work, we investigate the growth and transport mechanism of the SEI on Li and Na in contact with glyme- and carbonate-based electrolytes. The first electrolyte class is typically used in metal–sulfur and metal–oxygen cells, while the latter is a common class of electrolytes known in lithium-ion batteries since their commercialization in 1990s. EIS with small amplitudes (10 mV) is applied to investigate the growth mechanism under the conditions close to open-circuit potential. By combining various ex situ characterization techniques such as focused ion beam-scanning electron spectroscopy (FIB-SEM), XPS, and time-of-flight secondary ion mass spectroscopy (ToF-SIMS), the SEI growth behavior is inspected in more detail. The analysis of the activation energy is shown to be the key to obtaining mechanistic insights, although rarely done in battery research. Additionally, electrolyte properties such as the cation transference number and salt diffusion coefficient (*D*_salt_) were derived from the galvanostatic polarization method and low-frequency EIS, when possible.^28^

## 2 Experimental Section

### 2.1 Electrolyte and Electrode Preparation

Triethylene glycol dimethyl ether (triglyme, 99%) and molecular sieves (pore size of 3 Å, diameter of 1–2 mm) were purchased from Alfa Aesar. Ethylene carbonate (EC, 98%) and dimethyl carbonate (DMC, 98%) were purchased from Sigma-Aldrich. In order to remove the residual moisture, molecular sieves were first activated by heating to 180 °C under vacuum overnight and then added to the solvents. LiTf (LiCF_3_SO_3_, 98%, Sigma-Aldrich) and NaTf (NaCF_3_SO_3_, 99.5%, Solvionic) salts were dried prior to use (at 120 °C under vacuum, overnight). The moisture in the electrolytes was controlled to be under 20 ppm, as confirmed by Karl Fischer titration performed in an Ar-filled glovebox (atmosphere: O_2_ < 0.1 ppm, H_2_O < 0.1 ppm).

Due to the high reactivity of lithium and sodium metals, surface degradation is expected even in an Ar-filled glovebox. Thus, alkali metals used (Li rod with 99.9% trace metal basis and Na cubes containing mineral oil with 99.9% trace metals basis, both purchased from Sigma-Aldrich) were cut freshly each time right before the electrodes were prepared. Li and Na were subsequently sandwiched between two Celgard separators, roll-pressed to approximately the same thickness (0.15 mm), and then cut into disks with a diameter of 10 mm.

### 2.2 Cell Assembly

For electrochemical tests, CR2032-type coin cells made of stainless steel were assembled. Symmetric Li(Na) electrodes were attached to the stainless-steel disks with a diameter of 18 mm and were separated by a 20 μm thick Celgard separator (H2013). A constant amount of electrolyte (20 μL) was added to each cell.

### 2.3 Electrochemical Measurements

EIS was performed in the frequency range from 10^6^ to 0.01∼1 Hz, depending on the specific cells. The experiments were conducted in the potentiostatic mode, with an amplitude of 10 mV, using Solartron 1260 and Novocontrol Alpha-A devices. Temperature-dependent EIS measurements (in the range between 5 and 50 °C) were performed using an external thermostat (Lauda RC6CP). In this setup, two thermocouples were employed: one was placed in the vicinity of the coin cell for the precise sample temperature measurement and the other was located inside a water/oil bath for control purposes. Temperature-dependent EIS measurements were carried out for 30 min for each temperature (25 min to reach the equilibrium and 5 min for EIS measurement) to minimize the changes of the SEI during the experiment. Analysis of the impedance spectra was performed with ZView software (Scribner Associates, version 3.5c).

Galvanostatic polarization was performed using a Keithley Current Source (Model 220). Galvanostatic stripping–plating tests were performed in Li/electrolyte/Li and Li/electrolyte/Cu cells by a Neware Battery Testing System (BTS V.5.3 by Neware Technology Limited) with a constant current of 0.1 mA cm^–2^. Other parameters affecting the stripping–plating behavior such as the thickness of the electrode, the amount of liquid electrolyte, and the thickness of the separator were kept constant in every cell.

### 2.4 Morphological and Chemical Characterization of Surface Films

For FIB-SEM analyses, electrochemical coin cells were opened and electrodes were collected. Electrodes from the cells containing 1 M LiTf (NaTf) in triglyme and 1 M LiTf (NaTf) in EC/DMC = 50/50 (v/v) were washed with triglyme and EC/DMC = 50/50 (v/v), respectively, to avoid salt precipitation. Subsequently, they were dried under vacuum at room temperature overnight and transferred from an Ar-filled glovebox to a measurement chamber with self-made air-tight transfer tools. Cross-section images of the SEI on Li and Na metal electrodes were measured by a Zeiss Crossbeam scanning electron microscope with a built-in focused ion beam (FIB). FIB cutting was performed using a gallium beam (acceleration voltage: 30 kV) with current ranging from 200 pA to 2 nA, depending on the sample and its reactivity.

## 3 Results and Discussion

### 3.1 Impedances and Activation Energies: Li/Na Electrodes in Contact with the Salt-Free Solvent

The surface of Li/Na electrodes is expected to be covered by thin (several nanometers) layers of their native oxides, hydroxides, and carbonates.^29^ Even though thin layers might affect the electrolyte decomposition by differences in electron transfer,^30^ their Pilling–Bedworth ratios (the ratio of molar volume of the Li/Na SEI compounds to the molar volume of the metallic Li/Na, see Supporting Information, part XII, Table S2) suggest only partial coverage. As soon as the alkali metal comes in contact with liquid electrolyte, the SEI is formed on its surface, which is not only determined by the nature of the electrode but also by the composition of the liquid electrolyte.^31,32^ Typically, salt anions react with Li/Na to form inorganic compounds (e.g., MF, M_2_O, M_2_S, where M = Li and Na) on the surface of the electrode, while the solvent molecules tend to form organic SEI components such as polyolefins, semicarbonates, or even polymers.^31^

**Figure 1:**
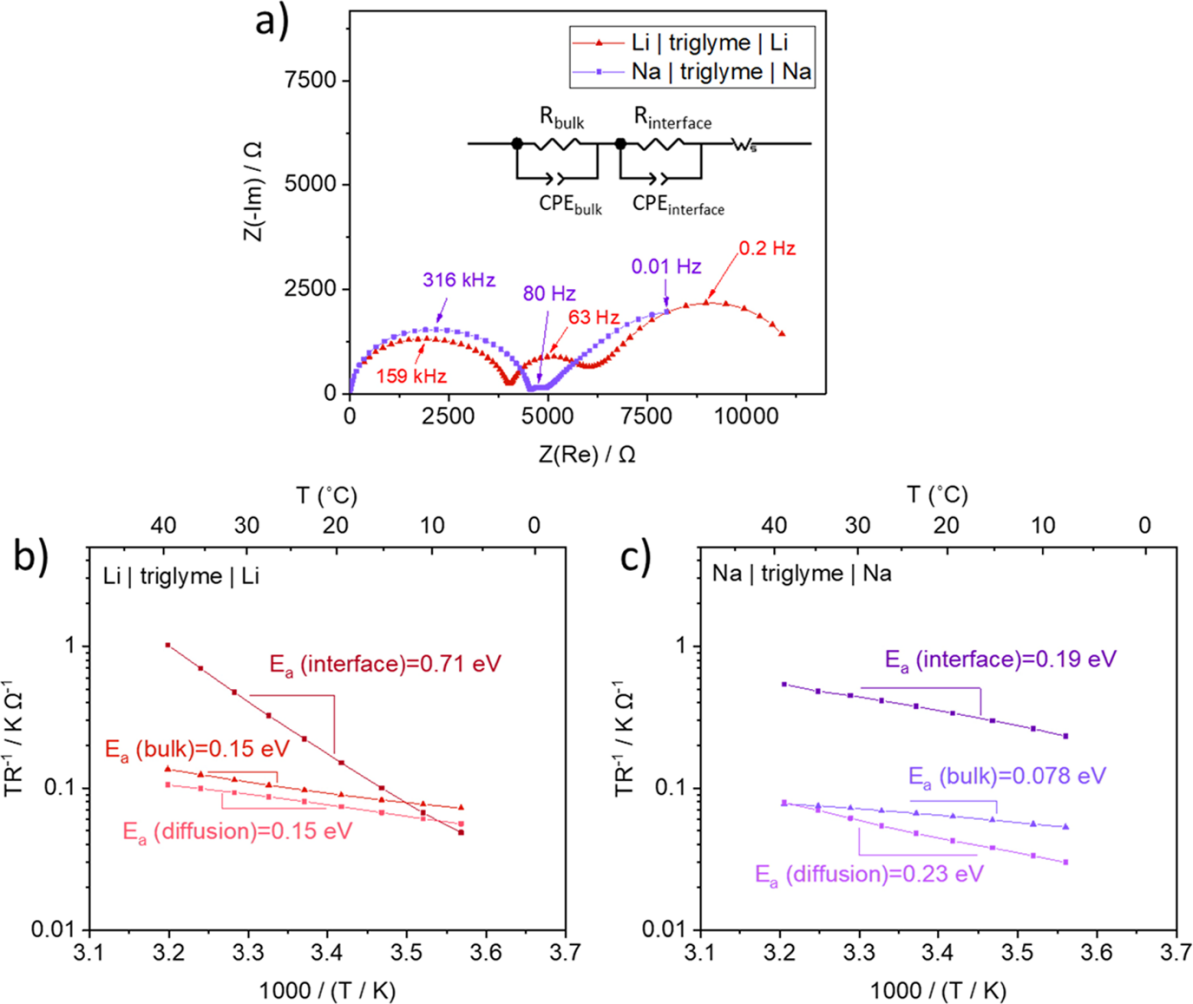
Investigation of symmetric Li/Na cells containing pure triglyme with EIS. (a) Nyquist plots with the characteristic frequency responses. The inset shows the corresponding equivalent circuit (*R*: resistance, CPE: constant phase element, *W*_s_: finite-length Warburg response with short circuit terminus). (b,c) Arrhenius plots and *E*_a_ of different contributions observed in (a) corresponding to bulk, interface, and Li(Na) diffusion. The cells were measured after 150 h of storage under open-circuit conditions.

First, symmetric Li/Na cells containing only triglyme solvent were tested for deconvolution of the contributions from the salt and those from the solvents. Nyquist plots in Figure 1a show three distinctive features contributing to the ionic transport. The semicircle that appears at the highest frequencies (hundreds of kilohertz) corresponds to the ionic transport in the electrolyte’s bulk (*R*_bulk_), since its capacitance is of the order of 10^–10^ F. The decrease of *R*_bulk_ with time (Supporting Information, Figure S1) was observed under open-circuit conditions, most probably a consequence of the distance decrease between two electrodes in a coin cell over time as confirmed by the experiment using a symmetric stainless-steel cell (ca. 60%, see Figure S2). Additionally, the *R*_bulk_ decrease could have been partially influenced by the dissolution of Li/Na from the electrode or SEI compounds, suggested by a small Li/Na concentration increase measured by inductively coupled plasma optical emission spectroscopy (ICP-OES) (see Table S1). The semicircle at medium frequency (tens of hertz) represents the ionic transport through the interfacial region, *R*_interface_ (equivalent to the *R*_SEI_ used later in the text), with the capacitance in the range of 10^–6^ F.^33^ As expected, *R*_interface_ changes over time (Figure S1) due to the continuous reaction between triglyme and Li/Na. The last semicircle at low frequency (lower than 1 Hz) shows a clear Warburg-type behavior, indicating concentration polarization of salt ions within the bulk liquid in the separator. Even though such polarization effects occur in the channels of the SEI (at low frequency), they are not expected to complicate the impedance arcs of relevance for our discussion.

The clear distinction of the three contributions enabled us to measure their activation energies (*E*_a_) by varying the cell temperature during the impedance measurement, giving further insight into their origin. Note that *E*_a_ was measured by lowering the temperature quickly (30 min for each data point) to exclude the effects of morphological or chemical changes of the SEI over time. For a symmetric Li-triglyme cell, identical *E*_a_ values were measured for the transport through bulk and polarization (0.15 eV), while the *E*_a_ of the interfacial contribution is found to be considerably higher (0.71 eV). The *E*_a_ value linked with the *R*_interface_ formed on Na is much lower (0.19 eV) and in between the values for bulk (0.08 eV) and cationic diffusion (0.23 eV). Generally speaking, the *E*_a_ values below 0.2 eV are a clear indication of liquid ion pathways without a significant series resistance, since *E*_a_ (bulk) of the electrolytes studied here is 0.15∼0.16 eV (see Figure S4).^34,35^ In summary, the results show that a more porous interface was formed in case of a Na-triglyme cell compared to a Li-triglyme cell, enabling ionic conduction through the liquid electrolyte permeated within the pores. The same measurements were performed for the case of salt-free Li-carbonate and Na-carbonate cells (see Figure S3), but a clear distinction between *R*_bulk_, *R*_interface_, and Warburg contribution was not possible due to the high total resistance amounting to MΩ.

### 3.2 SEI Growth and Transport in Li/Na Electrodes upon Contact with the Salt-in-Solvent Electrolyte

**Figure 2:**
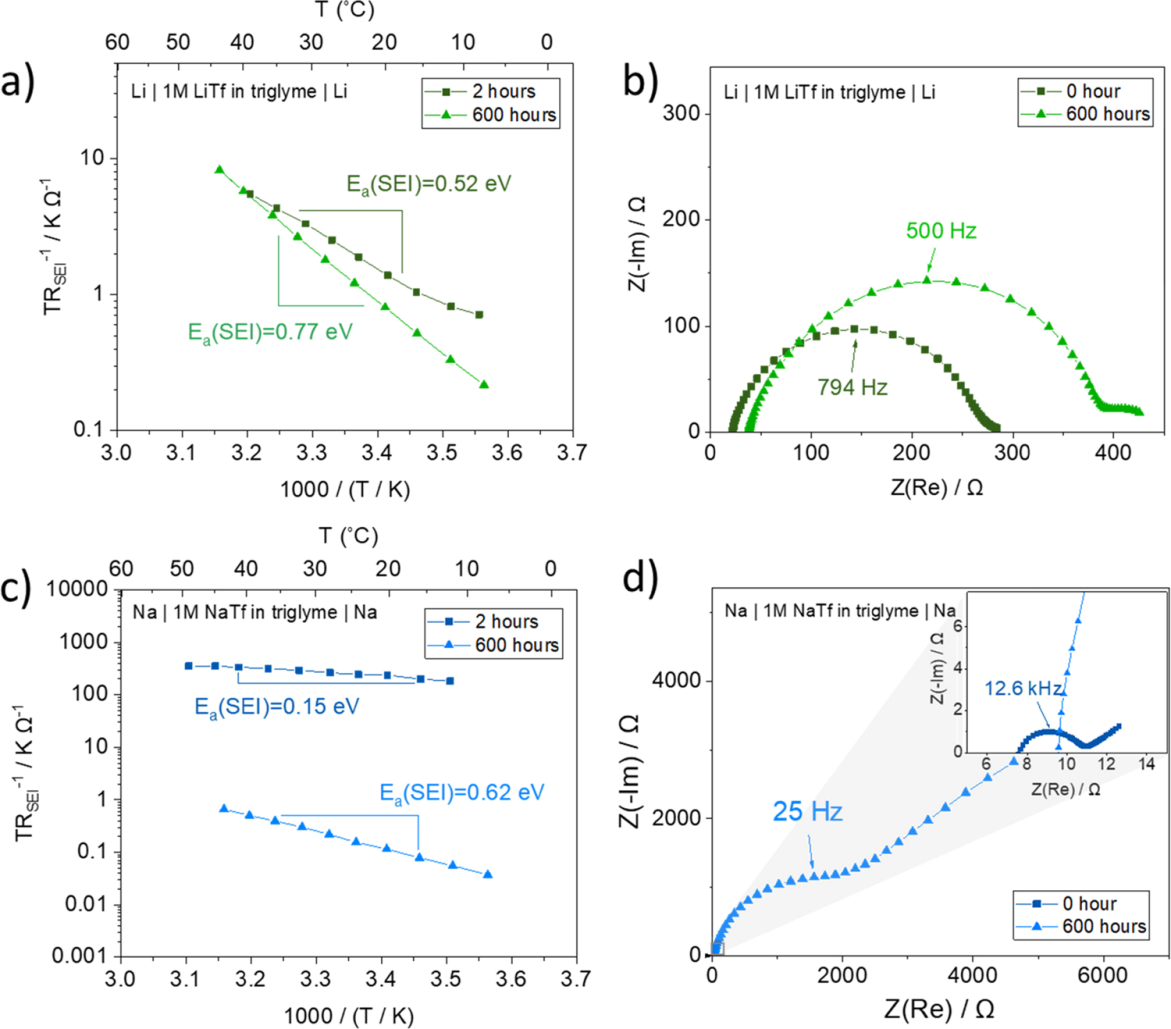
Evaluation of the changes in the activation energy of ion transport through SEIs. (a,c) Arrhenius plot of the Li(Na)-glyme system (symmetric Li or Na electrodes with 1 M LiTf or NaTf in triglyme) stored under open-circuit potential for 2 and 600 h. (b,d) Nyquist plots at room temperature before storage (0 h) and after storage (600 h) in the Li-glyme system and Na-glyme system, respectively.

For a better understanding of the ionic transport properties of SEIs, salt was added to the solvent and cells of Li-glyme (Li|1 M LiTf in triglyme|Li) and Na-glyme (Na|1 M NaTf in triglyme|Na) systems were assembled and aged upon open-circuit potential, followed by measurements of activation energies and SEI thicknesses. Figure 2a shows the evolution of the *E*_a_(SEI) over time in the case of the Li-glyme system, increasing from 0.52 to 0.77 eV after 600 h. Considering that inorganic Li SEI compounds show activation energies of ionic transport higher than 0.8 eV,^36−38^ an *E*_a_(SEI) value of 0.52 eV implies that the formed SEI is partially porous with ion transport pathways of solid and liquid phase mixtures. These values are in line with previous studies on LiClO_4_ and LiAsF_6_-containing electrolytes in contact with Li.^6^ An increased *E*_a_(SEI) of 0.77 eV could indicate the change of materials consisting SEIs. However, XPS surface analysis for SEIs on Li stored for 2 and 600 h under open-circuit conditions showed negligible change in the chemical composition of the SEI over time, and details can be found in the Supporting Information (Figures S8-1 and S8-2). Therefore, the increased value of *E*_a_(SEI) is most probably the result of densification, in other words, increase of the volume fraction of SEI’s solid phase. In the case of the Na-glyme system shown in Figure 2c, an exceptionally low *E*_a_(SEI) (0.15 eV) is observed at the very initial stage of storage, which is identical to the measured *E*_a_ of ion diffusion in the bulk liquid (Figure S4). Along with a strikingly small value of *E*_a_(SEI), an SEI resistance of 3 Ω (Figure 2d, inset) indicates that the SEI in this system is highly porous at the early stages of its growth, similarly as in the case of Na-triglyme without salt (Figure 1c). Such porous SEI allows liquid electrolyte to penetrate through it, resulting in low resistance of a liquid/solid composite at the beginning of the SEI growth. However, the liquid electrolyte continuously reacts with Na on the exposed surfaces, finally resulting in a denser and more resistive SEI as proven by higher *E*_a_(SEI) (0.62 eV) (Figure 2c) and higher *R*_SEI_ (>2000 Ω) (Figure 2d).

**Figure 3:**
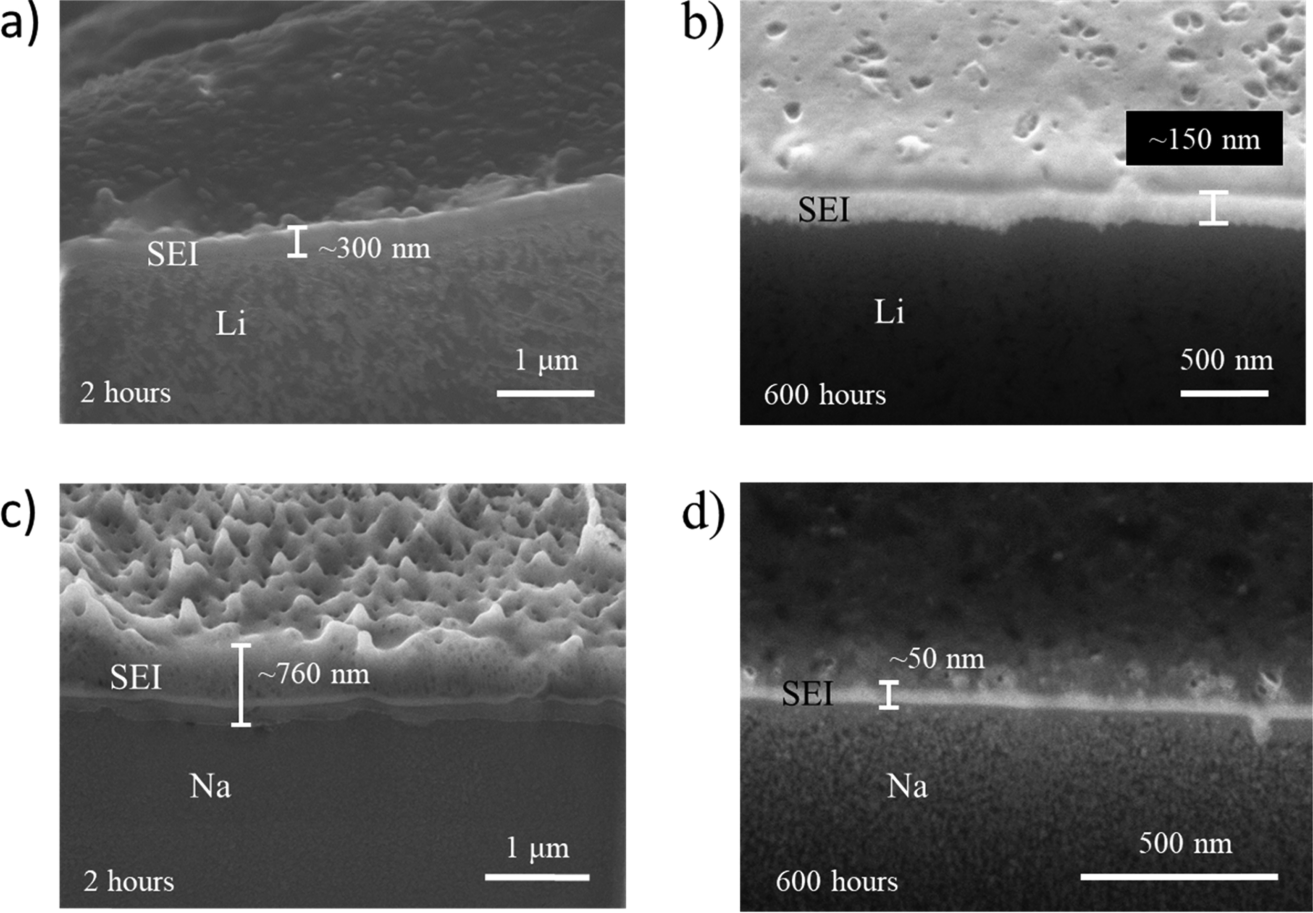
Cross-section images of SEIs on Li (a,b) and Na (c,d) observed by FIB-SEM. SEIs are formed by the contact between Li/Na and glyme-based electrolyte (1 M LiTf/NaTf in triglyme) and stored for 2 and 600 h.

Figure 3 shows cross-section images of SEIs on Li and Na formed by the contact with glyme-based electrolytes. A decrease in SEI thickness (300 nm to 150 nm) is evident in the Li-glyme system, indicating decreased ionic conductivity of the SEI at the end of aging, given by the increased SEI resistance (Figure 2b). On the contrary, initial porosity in the Na-glyme system is observed (Figure 3c), followed by a thinner and more compact SEI after 600 h of storage (Figure 3d). Considering the fact that increased *E*_a_(SEI) over time was observed for both Li-glyme and Na-glyme systems, the observed morphology and thickness of SEIs imply the densification (or compaction) of the SEIs. Note that the creep due to the pressure buildup in the cell causes the smoother surface of Na upon longer open-circuit potential storage.^39−41^

Similarly as glymes, carbonate-based electrolytes were in contact with Li/Na under open-circuit potential. The extracted *E*_a_(SEI) before and after long open-circuit storage, corresponding cross-section images, and surface analyses of Li with XPS are available in the Supporting Information (details can be found in Supporting Information Figures S5, S6, S8-3, and S8-4). Densification of the SEI is observed for Li-carbonate and Na-carbonate systems (supported by the increase of *E*_a_(SEI) in Figure S5) as well, and a porous SEI in the case of Na is evident from FIB-SEM (see Figure S6). For both glyme-based electrolyte and carbonate-based electrolyte cases, *E*_a_(SEI) on Na is lower than *E*_a_(SEI) of Li even after storage under open-circuit conditions for 600 h, implying that the SEI on Na is more porous than the SEI on Li. This claim is additionally supported by ToF-SIMS analyses of SEIs on Li/Na in the Supporting Information (Figure S7), where a clear distinction in roughness between Li and Na electrodes is shown. If the SEI is porous, electrolyte will permeate into the pores and upon electrode drying needed for sample preparation, only the solvent will evaporate, leaving the salt precipitated in the pores. Therefore, existence of the salt in the depth of the SEI is a clear indication of a porous SEI. For both Li-glyme and Li-carbonate systems (see Figure S7a,b), measured intensity of the salt anion (CF_3_SO_3_^–^) decreases approximately three orders of magnitude (∼0.001%, normalized intensity) after 600 s of sputtering. On the contrary, Na systems exhibit a significant amount of salt anion intensity (>0.1%) over 600 s of sputtering, implying the existence of salt in the bulk of the SEIs (see Figure S7c,d) and the porosity of SEIs. Note that here the sputtering time is not proportional to the sample depth and intensity is not directly proportional to the concentration of CF_3_SO_3_^–^, since both parameters are dependent on the surface roughness, element type, and the matrix surrounding the element.

In the summary of the results of electrochemical (EIS), chemical (XPS and ToF-SIMS) and morphological (FIB-SEM) characterizations in four different Li/Na systems (Li-glyme, Li-carbonate, Na-glyme, and Na-carbonate), it is concluded that (i) SEIs become denser over time in both Li/Na systems and (ii) SEIs on Na are more porous compared to the SEIs on Li, leading to the continuous SEI growth, which is not over even after long-term aging. However, in the case when *E*_a_ > 0.5 is observed, we cannot exclude the possibility that the activation energy is affected by space charge layer formation between different SEI phases, suggested in recent theoretical studies.^42^ For the special case of the initially formed (after 2 h) SEI on Na upon contact with glyme-based electrolytes, the *E*_a_ = 0.15 clearly indicates a porous SEI with predominant liquid pathways. Such low values of *E*_a_ are not possible for space charge zones as they always also contain additional migration contribution. We ascribe the higher porosity of SEIs on Na to the fact that the Pilling–Bedworth ratio of the expected inorganic Na SEI compounds is always lower than 1 (Table S2). On the other hand, several Li inorganic SEI compounds have an *R*_PB_ higher than 1, such as Li_2_CO_3_ (1.35), LiOH (1.26), and Li_2_S (1.06), enabling SEIs of higher density. However, this picture is simplified and *R*_PB_ of organic SEI compounds should not be overlooked, once their exact molecular structure and composition are known.

### 3.3 Determination of the Salt Diffusion Coefficients and Cationic Transference Number of Salt-in-Solvent Electrolytes

Bulk properties of the liquid SEI such as *t*_Li(Na)_ and *D*_salt_ were determined by the galvanostatic polarization method and the summary of results is shown in Table 1. Not only the transport behavior of SEIs but also the transport properties of electrolytes are of importance during stripping–plating, since they are directly correlated with Sand’s time (i.e., characteristic time when salt concentration at the surface decreases to zero causing the dendrite to start growing).^43^ Compared to Li, the transference number of Na is considerably higher in triglyme, which could be correlated either with the size of the ion and its higher mobility (consequence of the fact that Na is less solvated^44^) or to the specific molecular structure favoring positively charged ion aggregates containing Na. *t*_Na_ of carbonate-based electrolyte was difficult to be determined due to the high SEI resistance, making *R*_SEI_ and diffusion contribution not distinguishable (details can be found in Supporting Information part IX and Figure S9).

**Table 1:** Summary of the Cation Transference Numbers and Salt Diffusion Coefficients Derived from the Galvanostatic Polarization Method (Supporting Information, Figure S9)^a^

electrolytes	*t*_pol_	*D*_salt_ (cm^2^ s^–1^)
1M LiTf in triglyme	0.24	1.0 × 10^–7^
1M LiTf in EC/DMC	0.12	2.8 × 10^–6^
1M NaTf in triglyme	0.42 (*t*_EIS_ = 0.36)	1.7 × 10^–6^
1M NaTf in EC/DMC	N/A	5.8 × 10^–8^

a *t*_EIS_: the transference number measured by the low-frequency EIS method (Supporting Information, Figure S10).

### 3.4 Stripping–Plating Behavior after Open-Circuit Aging of Li/Na Cells

**Figure 4:**
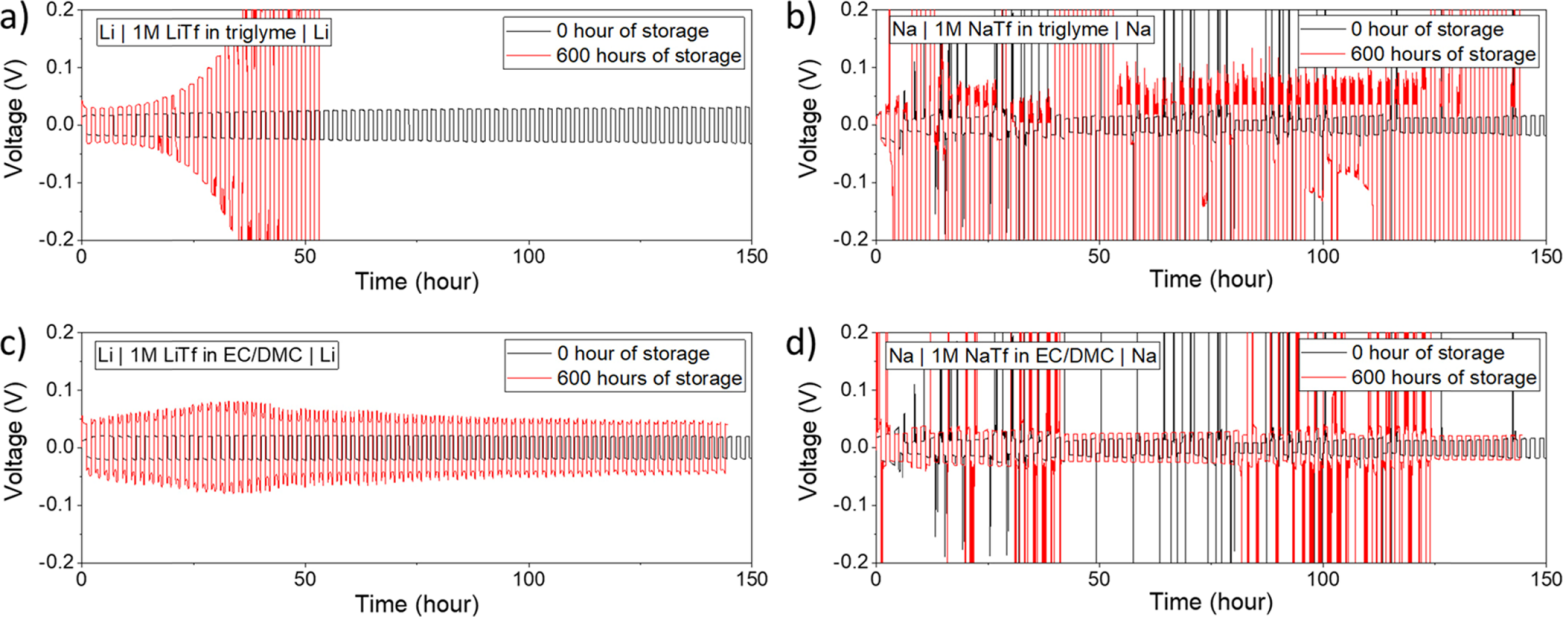
Stripping–plating behavior of symmetric Li/Na cells after initial cell assembly vs after 600 h of storage (applied current: 0.1 mA cm^–2^) with (a,b) triglyme and (c,d) carbonate electrolytes. Top-view images of the electrode after stripping–plating measured by SEM are shown in Figure S11.

The cyclic stripping–plating behavior was measured after aging under open-circuit potential directly after the cell assembly vs after 600 h of storage to investigate the overall performance of electrodes with SEIs formed in four different systems (Li-glyme, Na-glyme, Li-carbonate, and Na-carbonate). Figure 4 shows stripping–plating results of four symmetric cells where 0.1 mA cm^–2^ of constant current was applied for charging–discharging for 1 h each. In every case, stripping–plating behavior directly upon cell assembly (Figure 4, black) shows a more stable cycling behavior and lower overpotential compared to the cells stored for a long time (Figure 4, red). Indeed, the denser SEIs are beneficial for preventing further electrolyte decomposition but make the ion transport less facile as proven by the increased *E*_a_, resulting in larger overpotential and cell failure. Comparing Li and Na behavior in an overall manner, Na exhibits more frequent intermittent overpotential spikes whereas Li shows a gradual change of overpotential during cycling. This may be attributable to (i) chemically/mechanically unstable SEIs on Na^25−27^ and (ii) relatively low values of *D*_salt_ of the Na-salt-containing electrolyte, shortening Sand’s time.^45^ However, many other factors affecting cycling behavior such as chemical/morphological homogeneity and mechanical properties of the SEIs should also be considered, which could be examined systematically with atomic force microscopy as it is recently shown in the literature.^46−49^ Furthermore, it may not be neglected that stripping causes additional porosity or isolated metallic Li,^50,51^ leading to altered Li^+^/Na^+^ transport properties during galvanostatic cycling. The top-view SEM images of the surface of the corresponding electrodes after stripping–plating are available in the Supporting Information (see Figure S11). No direct correlation between cycling behavior and morphology is observed.

## 4 Conclusions

We investigated the long-term (∼a month) growth of SEIs on Li/Na metal electrodes with various nonaqueous liquid electrolytes under open-circuit potential and examined their ionic transport behavior over time. Glyme- and carbonate-based electrolytes were tested and compared in symmetric Li/Na cells. In every tested cell, SEIs densify over time (volume % increase of the solid phase on the account of the liquid phase) as confirmed by the increase of activation energy of the ionic transport (*E*_a_). We propose this type of evaluation as a standard method to estimate the porosity of the SEI. Compared to Li, Na is found to form more porous SEIs as confirmed by lower *E*_a_ and the presence of salt anions in the bulk measured by ToF-SIMS. The porous SEI appears to have low SEI resistance at the initial stage of storage but leads to the continuous growth of the interface through densification process. Therefore, the porosity of the SEI should be carefully taken into account for the SEIs formed in contact with liquid/solid electrolytes, especially for the case of Na where porosity is unavoidable by the nature of the inorganic phases in the formed film (*R*_PB_ < 1). Likewise, it is highly recommended to focus on the thorough experimental investigation and control of porosity of artificially prepared SEIs on alkali metal electrodes. We do not exclude the possibility that the differences in the native film formation between Li and Na strongly affect the differences in the formed SEI, but examining it will require experimentally challenging and persistent efforts. Additionally, we observe for the first time a considerably higher salt diffusion coefficient and cation transference number in Na-glyme electrolytes compared to their lithium counterparts.

## Supporting Information Available

EIS results of symmetric Li/Na cells and symmetric stainless-steel cells with pure glyme solvents; ICP-OES results of pure triglyme solvent in contact with Li/Na; EIS results of Li/Na symmetric cells with pure carbonate solvent; *E*_a_ of ion transport in bulk electrolytes; time-dependent *E*_a_ of Li-carbonate and Na-carbonate systems; FIB-SEM images of SEIs on Li and Na in contact with carbonate-based electrolyte for 2 and 600 h; ToF-SIMS spectra of SEIs on Li and Na in contact with glyme-/carbonate-based electrolytes after 600 h of storage under open-circuit conditions; XPS spectra of SEIs on Li in contact with glyme-/carbonate-based electrolytes; determination of the cation transference number (*t*_Li/Na_) and salt diffusion coefficient (*D*_salt_) by the galvanostatic polarization method; *t*_Na_ determination by the Sørensen and Jacobsen method; top-view SEM images of Li/Na electrodes after stripping/plating with glyme-/carbonate-based electrolyte stored for 0 vs 600 h; comparison of the Pilling–Bedworth ratio (*R*_PB_) of inorganic Li SEI compounds vs Na SEI compounds (PDF)
