# Supplementary material for: Porosity of Solid Electrolyte Interphases on Alkali Metal Electrodes with Liquid Electrolytes
Source: ACS Appl Mater Interfaces. 2021 Oct 20;13(43):51767–74. doi: 10.1021/acsami.1c15607 (PMC8569679; doi:10.1021/acsami.1c15607)
Supplement: Supplementary file 1 — am1c15607_si_001.pdf [file am1c15607_si_001.pdf]

Supporting information

Porosity of solid electrolyte interphases on alkali metal electrodes with liquid electrolytes

Kyungmi Lim, Bernhard Fenk, Jelena Popovic*, Joachim Maier

Max Planck Institute for Solid State Research, Stuttgart, Baden-Württemberg, Germany

Table of contents

I. EIS results of symmetric Li/Na cells and symmetric stainless steel cell with pure glyme solvents

II. ICP-OES results of pure triglyme solvent contacted with Li/Na

III. EIS results of Li/Na symmetric cells with pure carbonate solvent

IV. Activation energy (E_a) of ion transport in bulk electrolytes

V. Time-dependent E_a of Li-carbonate and Na-carbonate systems

VI. FIB-SEM images of SEIs on Li and Na contacted with carbonate-based electrolyte for 2 hours and 600 hours

VII. ToF-SIMS results of SEIs on Li and Na contacted with glyme-/carbonated-based electrolytes after 600 hours of storage under open-circuit condition

VIII. XPS results of SEIs on Li contacted with glyme-/carbonate-based electrolytes

IX. Determination of cation transference number ($t_{Li/Na}$) and salt diffusion coefficient (D_{salt}) by galvanostatic polarization method

X. t_{Na} determination by Sørensen and Jacobsen method

XI. Top-view SEM images of Li/Na electrodes after stripping/plating with glyme-/carbonate-based electrolyte stored for 0 hour *vs*. 600 hours

XII. Comparison of Pilling-Bedworth ratio (R_{PB}) of Li SEI compounds *vs*. Na SEI compounds

Ⅰ. EIS results of symmetric Li/Na cells and symmetric stainless steel cell with pure glyme solvents

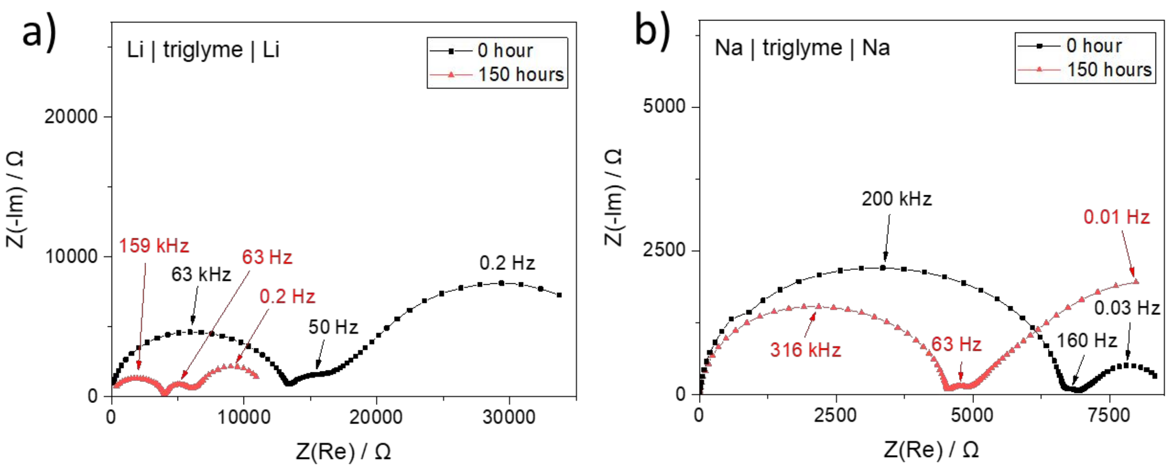

Figure S1. Nyquist plots of Li (a) and Na (b) symmetric cell in contact with pure triglyme solvent and their evolution over time.

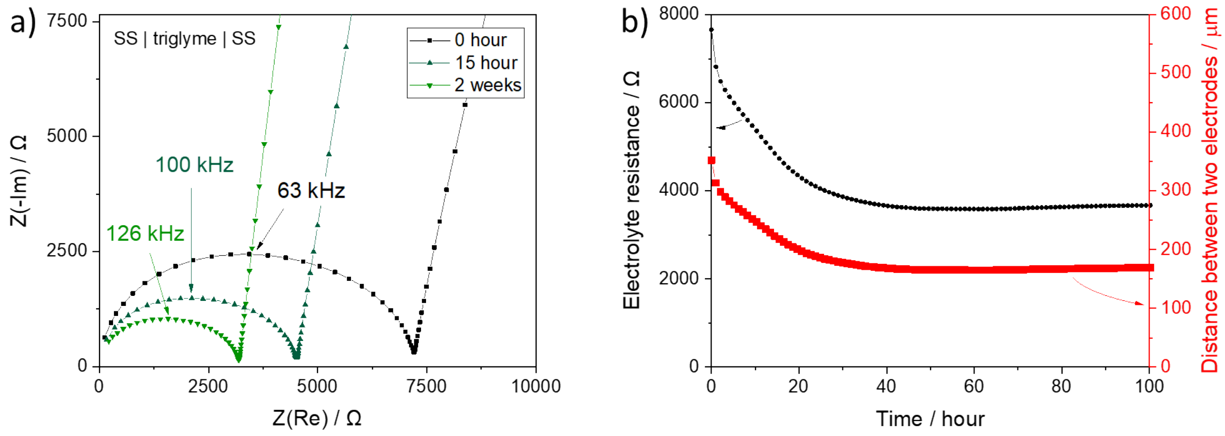

Figure S2. (a) Nyquist plots of stainless steel | triglyme | stainless steel symmetric cell. (b) Resistance and corresponding distance between two electrodes over time. The distance between two electrodes was derived by $L = \sigma RA$ (L: distance, σ: ionic conductivity of triglyme, R: resistance measured in (a), A: surface area of the electrode)

II. ICP-OES results of pure triglyme solvent contacted with Li/Na

Inductively coupled plasma optical emission spectroscopy (ICP-OES) was done with Spectro Ciros device. Liquid samples were diluted in water in a ratio of 1:1000.

Table S1. Concentration of Li and Na measured by ICP-OES. For sample 2 and 3, pure triglyme was contacted with Li/Na metal for 150 hours. (Unit: mmol L^{-1})

Sample	Li	Na
1) Pure triglyme	--	6.09×10^{-4}
2) Pure triglyme contacted to Li metal	14.9	3.48×10^{-4}
3) Pure triglyme contacted to Na metal	2.88×10^{-4}	16.8

Ⅲ. EIS results of Li/Na symmetric cells with pure carbonate solvent

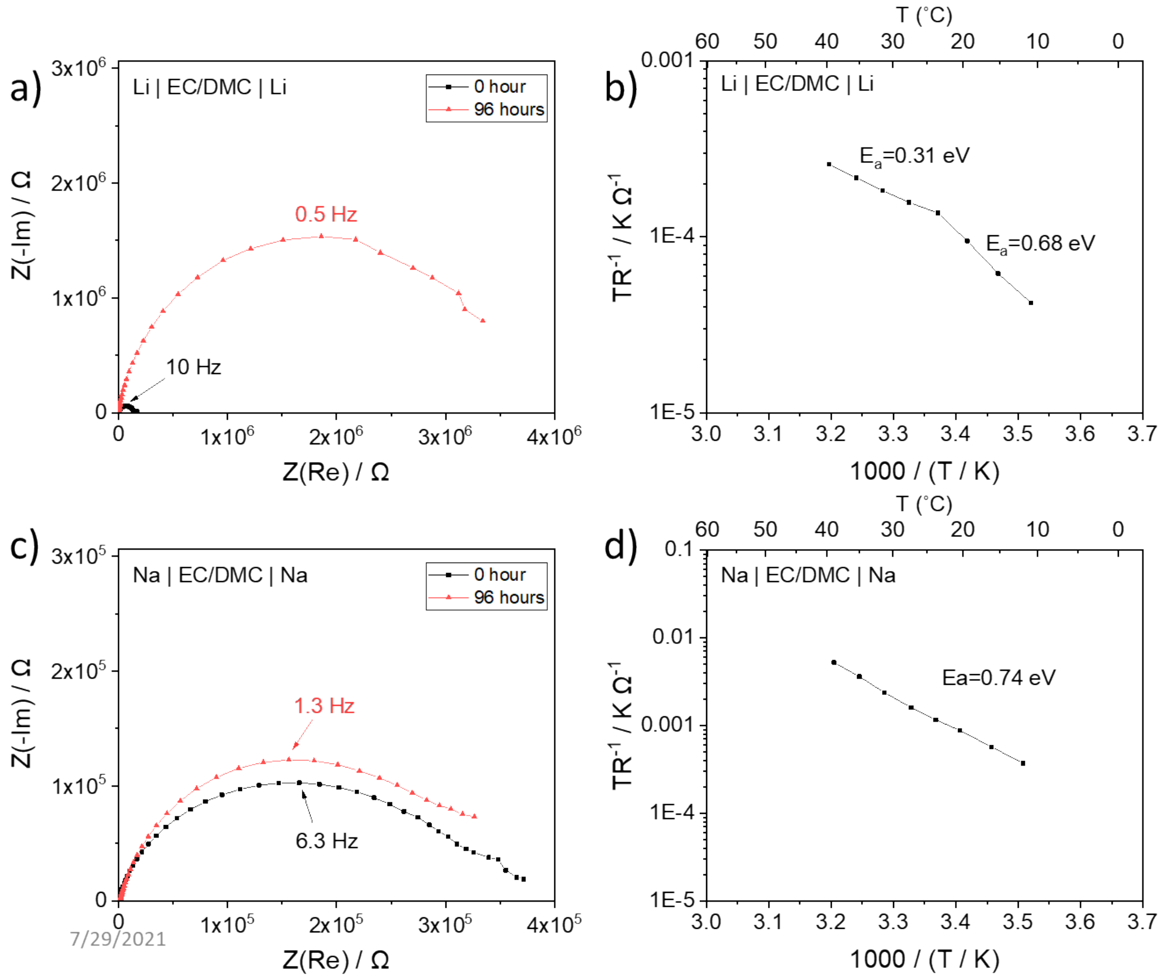

Figure S3. EIS investigation of Li/Na symmetric cells containing EC/DMC=50/50 (v/v) without salts. (a, c) Nyquist plots with characteristic frequency responses in Li, Na-carbonate system, respectively. (b, d) Arrhenius plots and E_a corresponding to (a, c).

Ⅳ. Activation energy (E_a) of ion transport in bulk electrolytes

For EIS measurement of liquid electrolytes with fixed electrode distance at room temperature, self-made vessel was used. The vessel consists of two electrodes made of gold-coated brass and a Teflon ring for adjustment of the electrode distance.

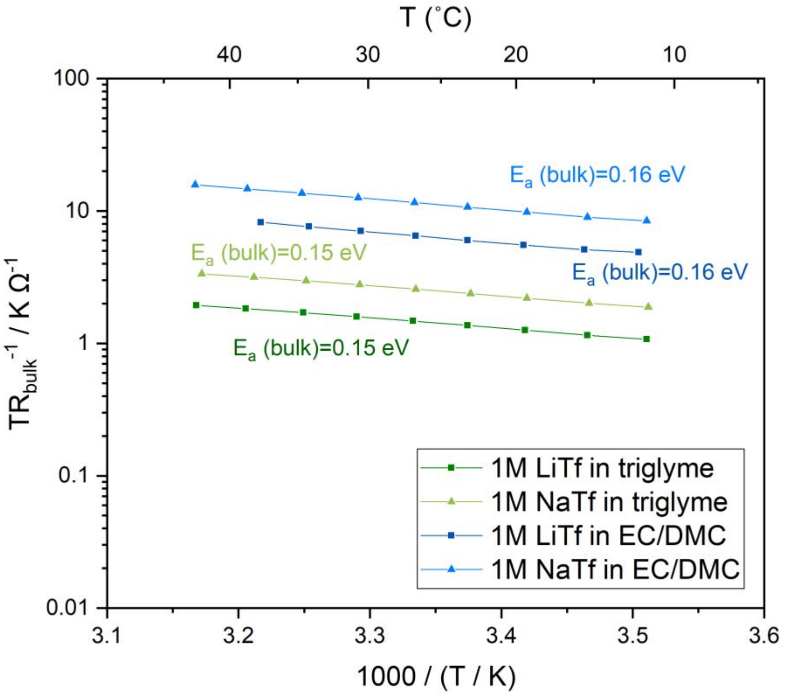

Figure S4. Temperature dependency of bulk electrolyte resistance and activation energy in four different electrolytes.

V. Time-dependent *Ea* of Li-carbonate and Na-carbonate systems

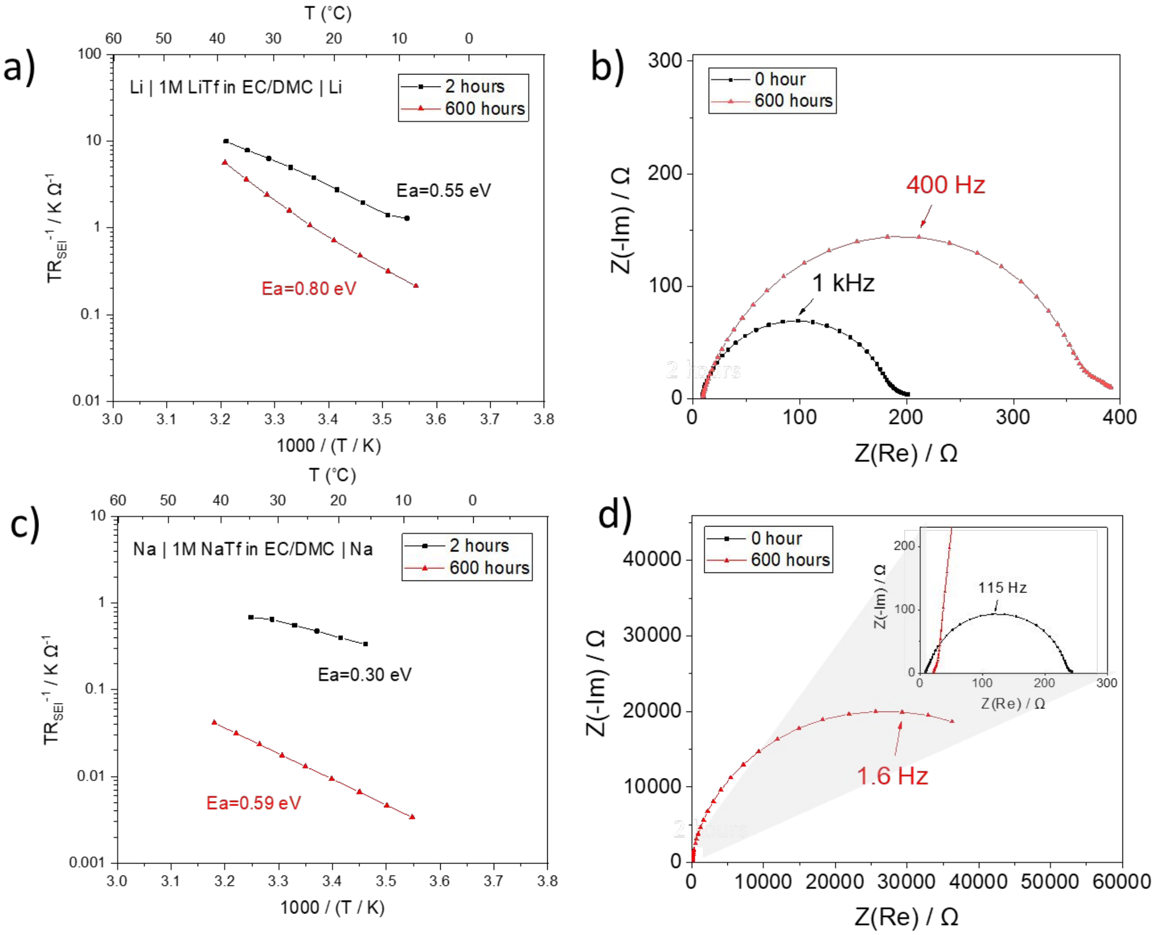

Figure S5. The evaluation of the changes in the activation energy of ion transport through SEIs. (a, c) Arrhenius plot of Li(Na)-carbonate system (symmetric Li or Na electrodes with 1M LiTf or NaTf in EC/DMC) stored under open-circuit condition for 2 hours and 600 hours. (b, d) show impedance spectra at room temperature before storage (0 hour) and after storage (600 hours) in Li-carbonate and Na-carbonate system, respectively.

Ⅵ. FIB-SEM images of SEIs on Li and Na contacted with carbonate-based electrolyte for 2 hours and 600 hours

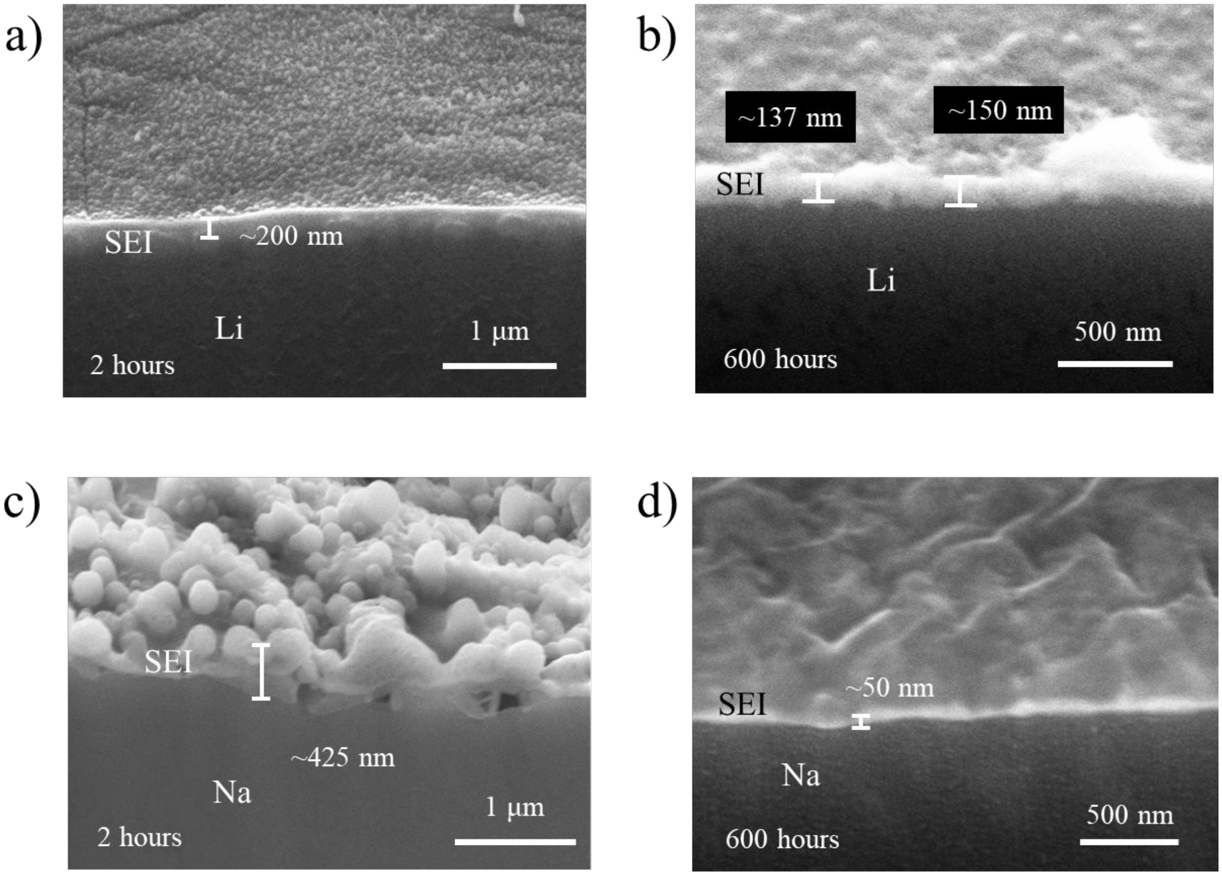

Figure S6. Cross-section images of SEIs on Li and Na measured by FIB-SEM. SEIs are formed by the contact between Li and carbonate-based electrolyte (1M LiTf in EC/DMC) and stored for (a) 2 hours and (b) 600 hours. Similarly, SEIs on Na are formed by the contact between Na and carbonate-based electrolyte (1M NaTf in EC/DMC) and stored for (c) 2 hours and (d) 600 hours.

Ⅶ. ToF-SIMS results of SEIs on Li and Na contacted with glyme-/carbonate-based electrolytes after 600 hours of storage under open-circuit condition

Sample preparation was done in the same manner as in the FIB-SEM analyses. ToF-SIMS measurements were carried out using mass spectrometer ToF-SIMS-CNS (IONTOF). Firstly, samples were sputtered with Cs-source sputter gun operated with an acceleration energy of 0.5 keV with current of 124~152 nA in the area of 300 × 300 μm^2. For analysis, Bi-source gun accelerated with the voltage of 30 kV and the beam current of 0.66 pA was used in the area of 50 × 50 μm^2. Analysis of ToF-SIMS data was done with software Surfacelab 7.1.

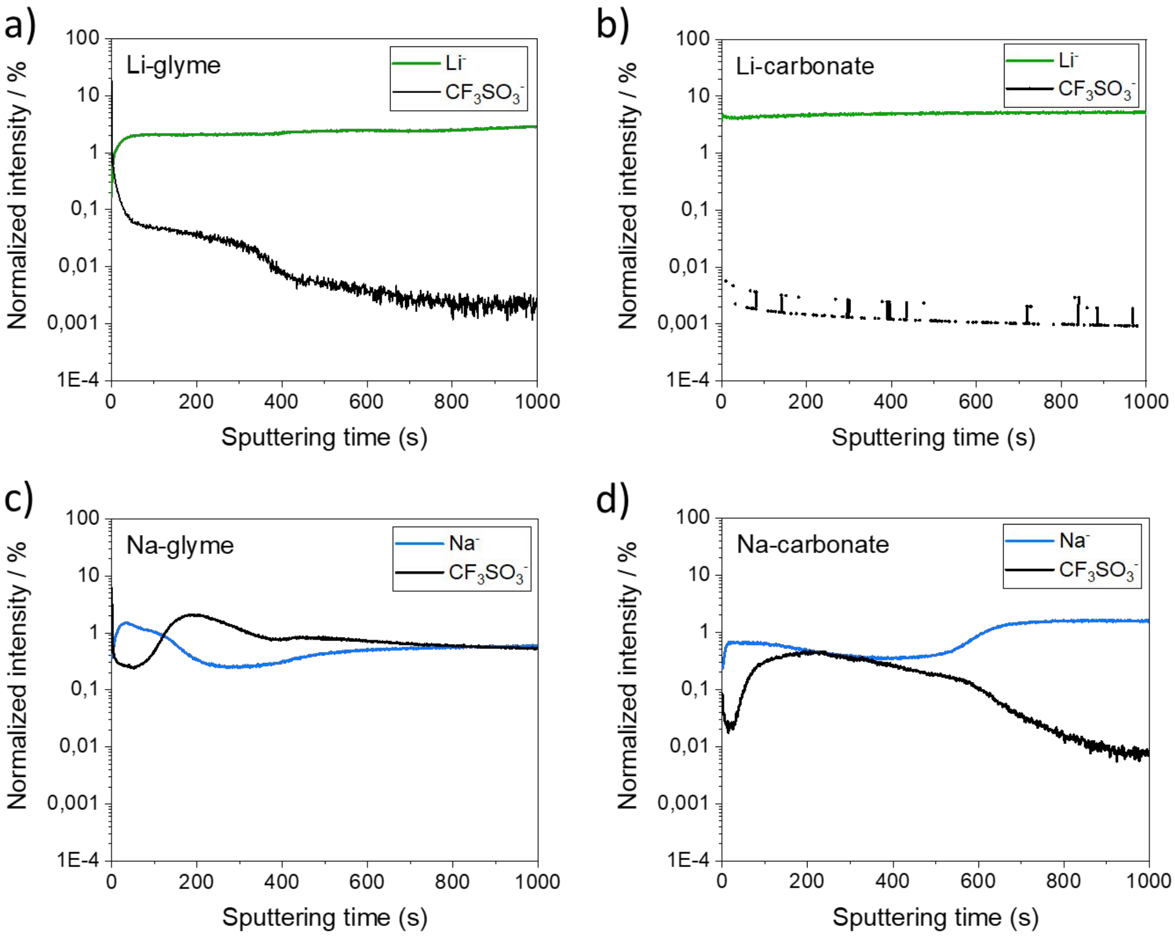

Figure S7. ToF-SIMS depth profiles of SEIs formed by contacting Li/Na with liquid electrolytes for 600 hours. (a) Li contacted with 1M LiTf in triglyme, (b) Li contacted with 1M LiTf in EC/DMC, (c) Na contacted with 1M NaTf in triglyme and (d) Na contacted with 1M NaTf in EC/DMC. Number of counts (y axis) were normalized by total intensity.

Ⅷ. XPS results of SEIs on Li contacted with glyme-/carbonate-based electrolytes

XPS was performed on a Kratos Axis Ultra system with a monochromatic Al Kα X-ray source. High-resolution data was acquired with a pass energy of 20 eV. Ar^+ sputtering was performed using a scanned Minibeam III sputter gun (Kratos) with a beam energy of 4 kV and an emission current of 20 mA. XPS data was analysed with CasaXPS software (version 2.3.23PR1.0 by Casa Software Ltd).

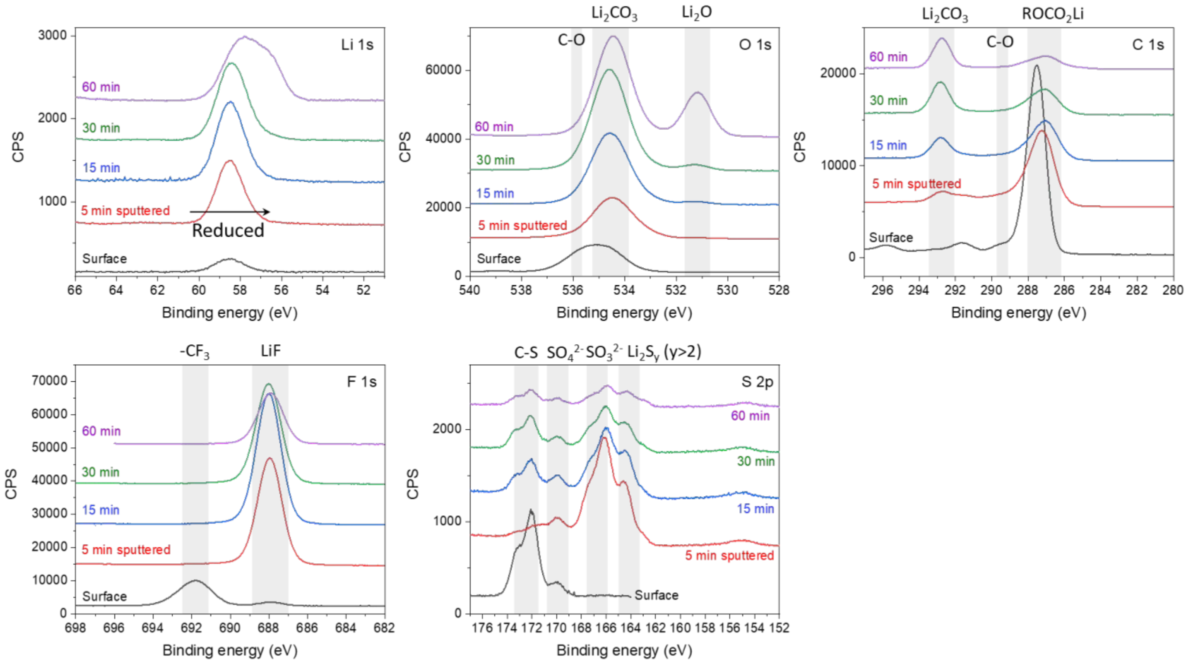

Figure S8-1. SEI on Li formed by contacting with 1M LiTf in triglyme for 2 hours

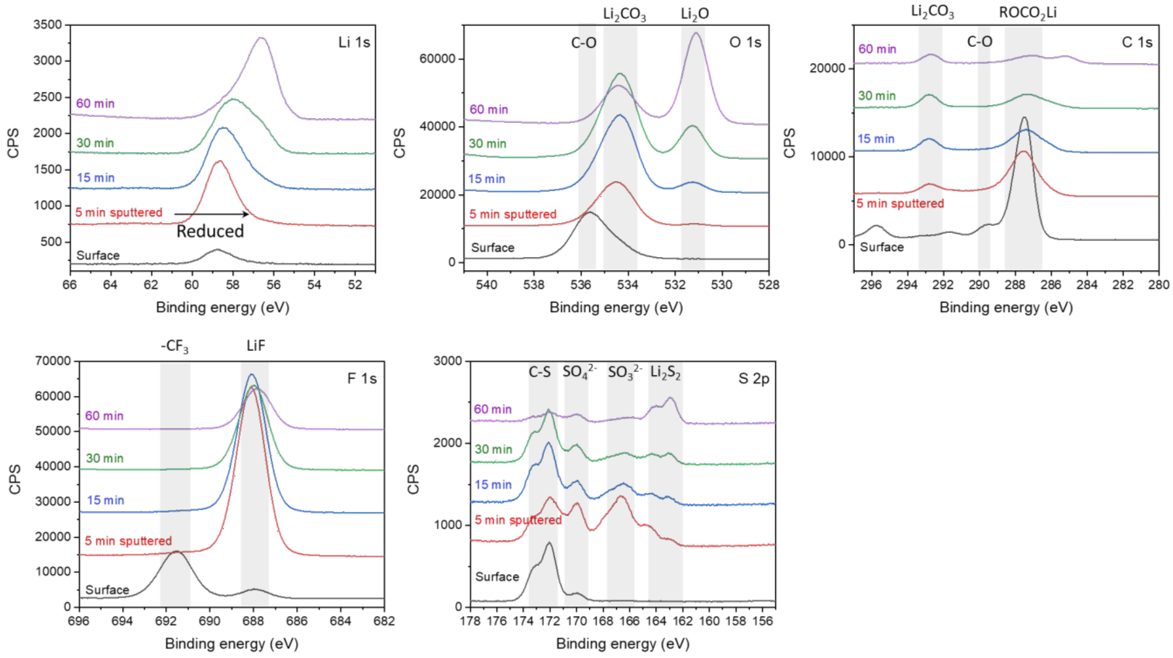

Figure S8-2. SEI on Li formed by contacting with 1M LiTf in triglyme for 600 hours

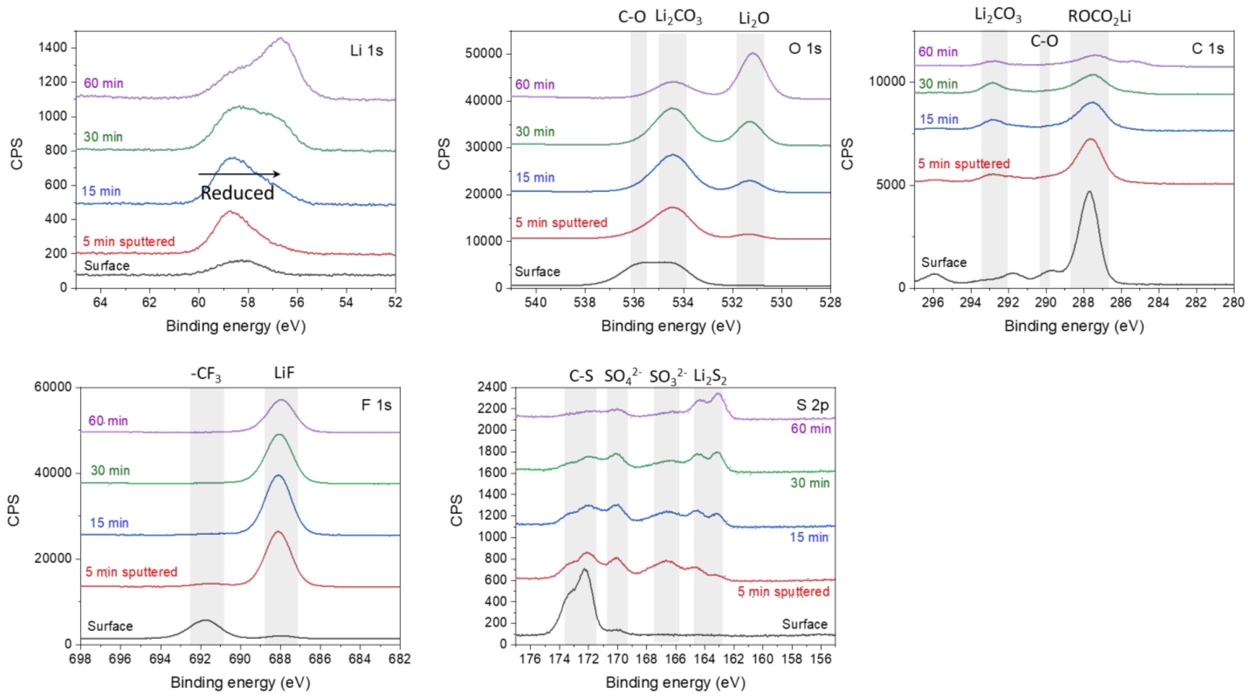

Figure S8-3. SEI on Li formed by contacting with 1M LiTf in EC/DMC for 2 hours.

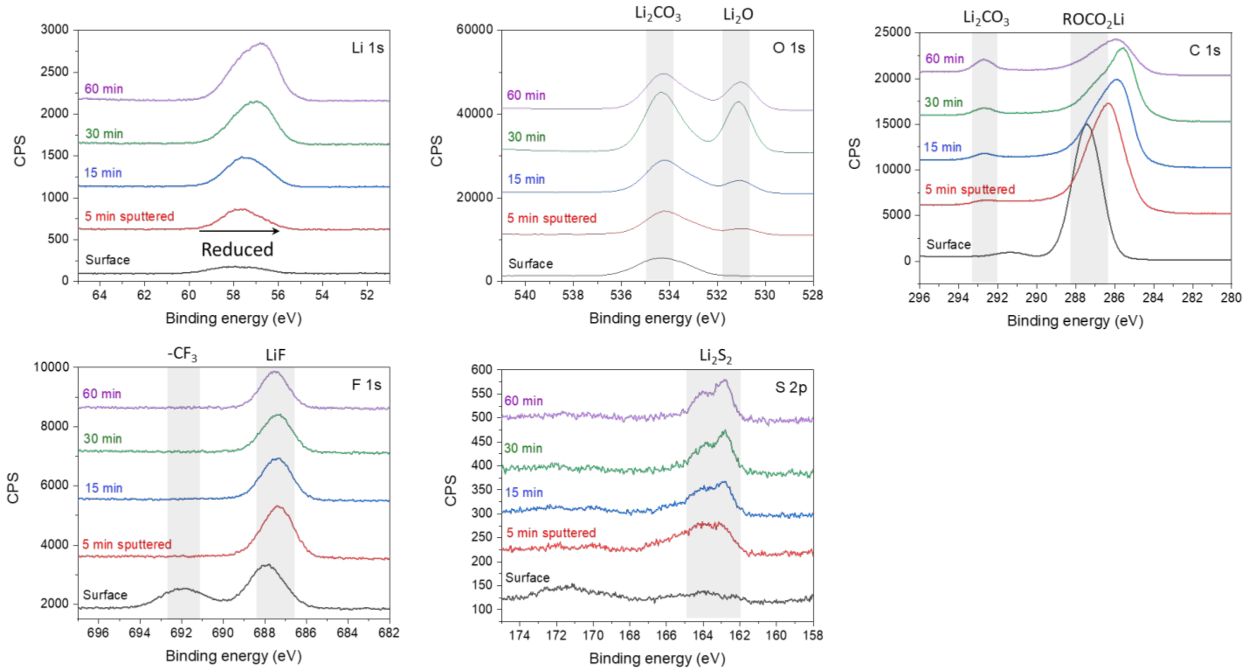

Figure S8-4. SEI on Li formed by contacting with 1M LiTf in EC/DMC for 600 hours.

IX. Determination of cation transference number ($t_{Li/Na}$) and salt diffusion coefficient (D_{salt}) by galvanostatic polarization method

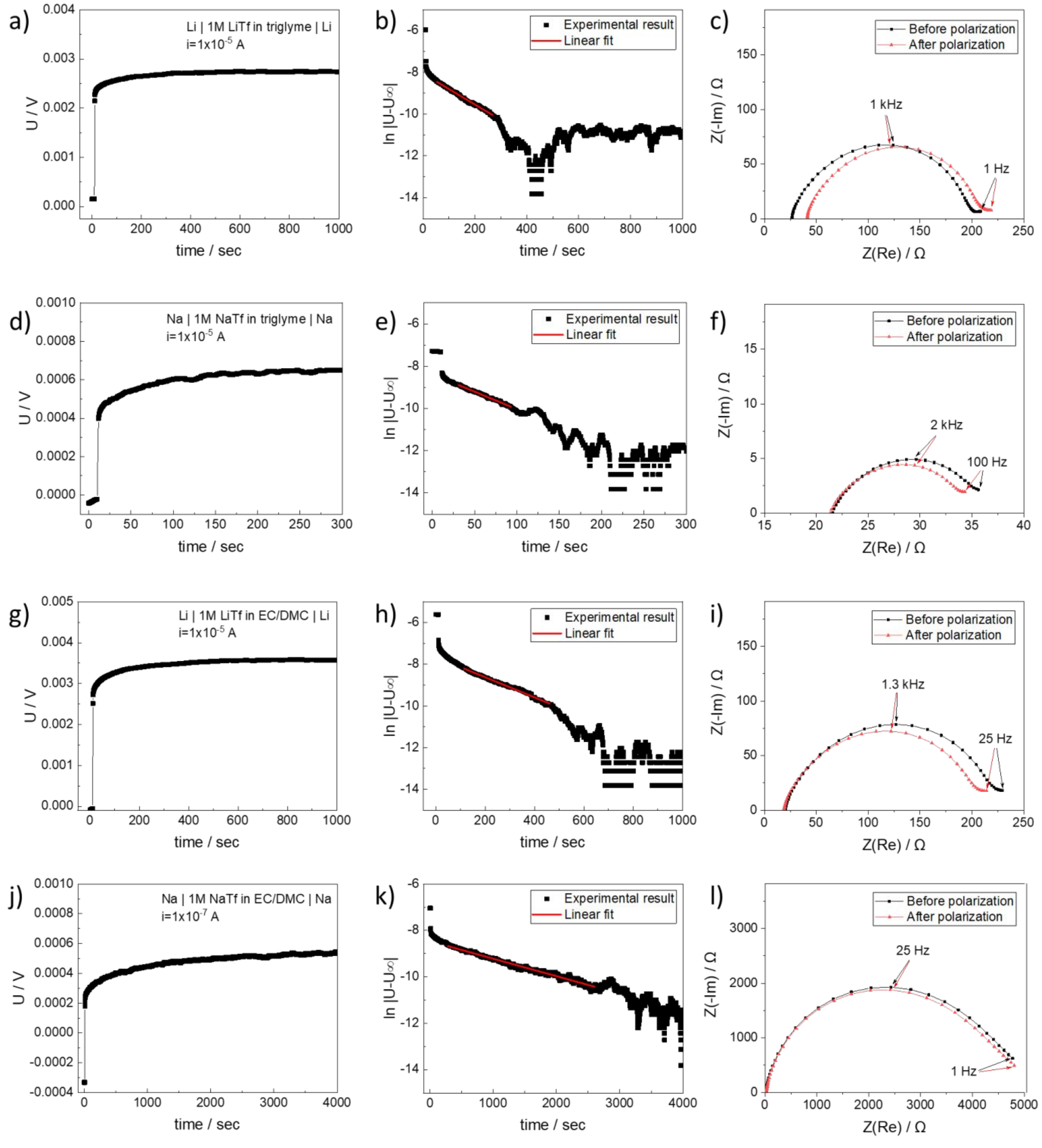

Figure S9. Determination of the cation transference number and salt diffusion coefficient by galvanostatic polarization method in (a-c) Li-glyme, (d-f) Na-glyme, (g-i) Li-carbonate and (j-l) Na-carbonate systems. (a, d, g, j) Time-dependent galvanostatic polarization curve. (b, e, h, k) Time *vs*. $\ln|U\text{-}U_{\infty}|$ curve derived from (a, d, g, j), respectively. (c, f, i, l) Nyquist plots corresponding to EIS measurements before/after galvanostatic polarization.

The cation transference number was derived from[1]

$$t_{pol} = \frac{I_0(R_{tot,0} - R_{SEI,0})}{U_\infty - I_0 R_{SEI,\infty}} \quad (1)$$

where t_{pol} is transference number measured by polarization method, I_0 is applied current, $R_{tot,0}$ is total resistance before polarization, $R_{SEI,0}$ is SEI resistance before polarization, U_∞ is steady-state voltage, $R_{SEI,\infty}$ is SEI resistance after polarization.

Salt diffusion coefficient (D_{salt}) was determined by the slope of time *vs*. ln$|U\text{-}U_\infty|$ curve (Fig. S8b, e, h, k) yielded by exponential law:

$$ln\,[U(t) - U(t=\infty)] = const \pm t/\tau^* \qquad (\tau^* = L^2/\pi D_{salt}) \quad (2)$$

where L is diffusion length, equivalent to the distance between two electrodes. L was estimated by

$$L = \sigma_{bulk} R_{bulk} A \quad (3)$$

where σ_{bulk} is ionic conductivity of electrolyte, R_{bulk} is electrolyte resistance measured from EIS and A is electrode surface area.

X. t_{Na} determination by Sørensen and Jacobsen method

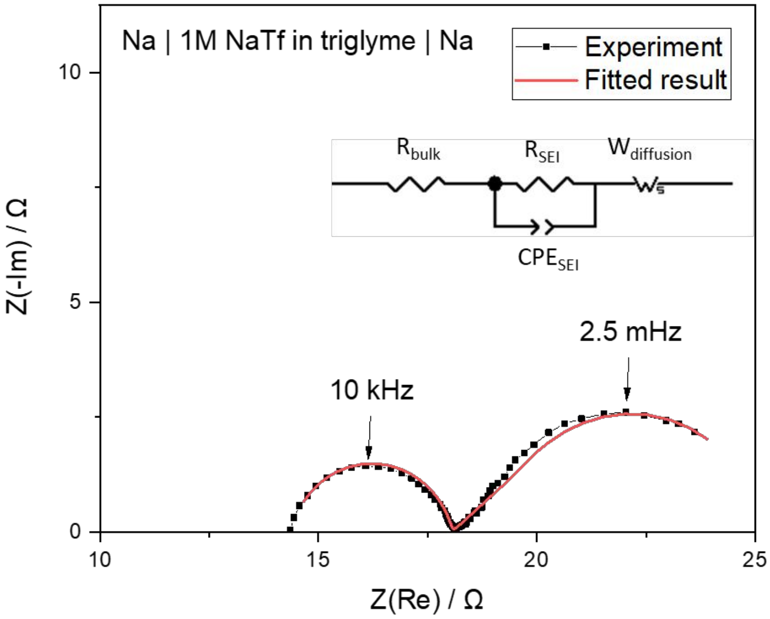

Figure S10. Impedance spectra of Na-glyme system measured in the frequency range of 10^{6}-10^{-3} Hz. Transference number was derived from the method suggested by Sørensen and Jacobsen.[2]

Transference number was calculated from

$$t_+ = \frac{1}{1 + \frac{Z_d(0)}{R_{bulk}}} = 0.364 \quad (4)$$

where $Z_d(0)$ is a diameter of low frequency arc and R_{bulk} is bulk electrolyte resistance.

Ⅺ. Top-view SEM images of Li/Na electrodes after stripping/plating with glyme-/carbonate-based electrolyte stored for 0 hour *vs*. 600 hours

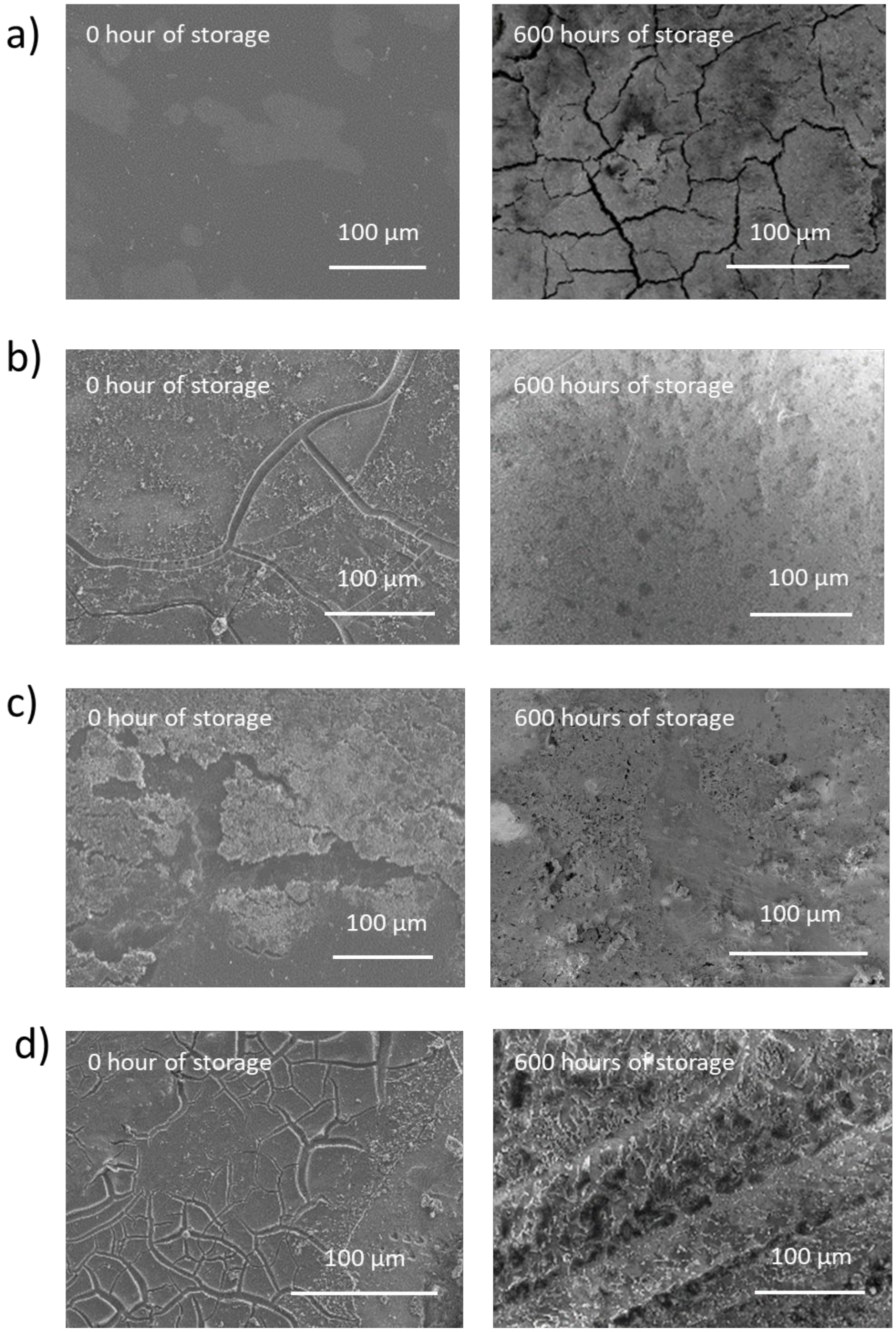

Figure S11. Top-view images of electrodes after stripping-plating in (a) Li-glyme, (b) Na-glyme, (c) Li-carbonate and (d) Na-carbonate systems.

Ⅻ. Comparison of Pilling-Bedworth ratio (R_{PB}) of Li SEI compounds *vs*. Na SEI compounds

Table S2. Comparison of Piling-Bedworth ratio (R_{PB}) of inorganic Li/Na SEI compounds.

Li SEI compounds		Na SEI compounds	
Compound name	R_{PB}	Compound name	R_{PB}
LiF	0.76	NaF	0.69
Li_2O	0.57	Na_2O	0.58
Li_2O_2	0.76	Na_2O_2	0.59
Li_2CO_3	1.35	Na_2CO_3	0.88
LiOH	1.26	NaOH	0.79
Li_2S	1.06	Na_2S	0.89
LiH	0.75	NaH	0.72

Pilling-Bedworth ratio (R_{PB}) is the ratio of molar volume of the Li compounds to the molar volume of the metallic Li. R_{PB} was calculated from

$$R_{PB} = \frac{V_{Li\ compound}}{n \times V_{Li}} \tag{5}$$

where $V_{Li\ compound}$ is the molar volume of lithium compound, n is number of reacted Li and V_{Li} is molar volume of metallic Li. Same equation was applied for Na.

References

(1) Evans, J.; Vincent, C. A.; Bruce, P. G. Electrochemical measurement of transference numbers in polymer electrolytes. *Polymer* **1987,** *28* (13), 2324-2328, DOI: https://doi.org/10.1016/0032-3861(87)90394-6.

(2) Ravn Sørensen, P.; Jacobsen, T. Conductivity, charge transfer and transport number—an ac-investigation of the polymer electrolyte LiSCN-poly(ethyleneoxide). *Electrochimica Acta* **1982,** *27* (12), 1671-1675, DOI: https://doi.org/10.1016/0013-4686(82)80162-X.
